# Ruptured gastro-intestinal stromal tumor as a surgical emergency: A case report and literature review

**DOI:** 10.1093/jscr/rjac434

**Published:** 2022-11-25

**Authors:** Mohamed Abdelgawad, Omar M Kamel, Peter P Issa, Mahmoud Omar, Lutfi Barghuthi, Tyler Davis, Hishaam Ismael

**Affiliations:** Department of Surgery, University of Texas Health Science Center, UT Health East Texas, Tyler, TX, USA; Department of Surgery, School of Medicine, Tulane University, New Orleans, LA, USA; School of Medicine, Louisiana State University Health Sciences Center, New Orleans, LA, USA; Department of Surgery, School of Medicine, Tulane University, New Orleans, LA, USA; Department of Surgery, University of Texas Health Science Center, UT Health East Texas, Tyler, TX, USA; Department of Surgery, University of Texas Health Science Center, UT Health East Texas, Tyler, TX, USA; Department of Surgery, University of Texas Health Science Center, UT Health East Texas, Tyler, TX, USA

**Keywords:** Gastrointestinal stromal tumor, GIST, Recurrence, Imatinib, Case Report

## Abstract

Gastrointestinal stromal tumors (GISTs) are the most common mesenchymal tumors of the gastrointestinal tract. GISTs of the small bowel are rare, and often present with an abdominal mass and/or bleeding. Chemotherapy and surgery are the mainstay of therapy. Here, we discuss an unusual case of a ruptured jejunal GIST with hemoperitoneum and recurrence despite surgical excision followed by Imatinib treatment. Forty-five cases of ruptured small intestinal GISTs were identified in the literature. Most cases were in males and were found to be at the site of the jejunum.

## INTRODUCTION

Gastrointestinal stromal tumors (GISTs) are the most common mesenchymal tumors of the gastrointestinal tract. Most commonly presenting during a patient’s sixth decade of life, GISTs represent between 0.1 and 3% of newly diagnosed gastrointestinal tumors [1]. GISTs develop along the length of the alimentary canal, including the stomach (60–70%), small intestine (20–25%), colon/rectum (5%) and esophagus (<5%). Tumors presenting beyond the gastrointestinal tract itself are rare [2]. Considering GISTs often have a mutation in the *KIT* gene, which encodes a proto-oncogene receptor tyrosine kinase, the use of Imatinib Mesylate, a chemotherapeutic agent targeting the tyrosine kinase c-kit, had been approved for treatment [3]. Following small bowel resection, Imatinib use improves overall survival and limits disease progression [4]. Imatinib may also downstage unresectable tumors to a resectable stage [5].

GISTs of the small intestine differ from those of gastric origin. While tumors of the small intestine are much less common, they are more often associated with the *c-kit* mutation. GISTs of the small intestine also rupture more frequently and are therefore more likely to undergo emergency surgery [6]. In addition, small intestinal GISTs more often have a mutated exon 9 (as opposed to gastric GISTs with mutated exon 11), which could explain why the latter generally responds more favorably to Imatinib and recurs less frequently [7]. Here, we present an interesting case of a ruptured mid-jejunal GIST with hemoperitoneum treated by surgical excision and Imatinib treatment which recurred within 2 months.

## CASE REPORT

An 81-year-old male with unremarkable past medical and surgical histories presented with a one-day history of acute-onset generalized abdominal pain, nausea and vomiting. The patient was vitally stable on physical examination, with notable abdominal tenderness. Initial lab work was within normal limits (Hb 12.4 g/dl).

**Figure 1:**
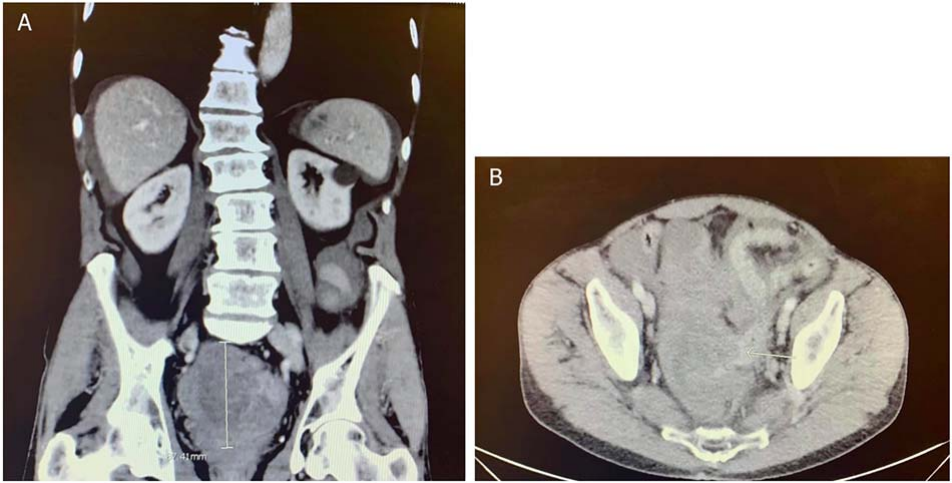
CT abdomen and pelvis with contrast in (**A**) anterior–posterior and (**B**) coronal views identified a rectovesical hematoma mass measuring 8.2 × 7 cm.

**Figure 2:**
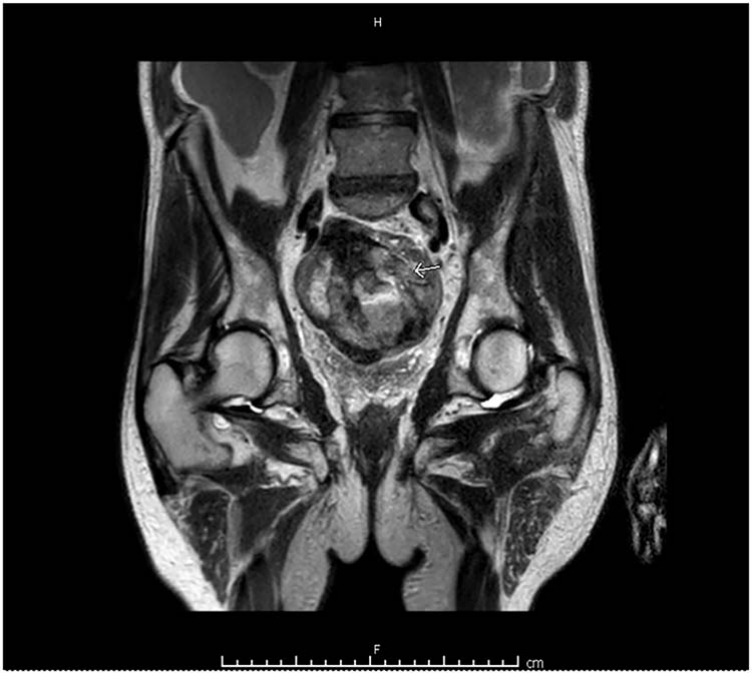
MRI abdomen and pelvis with contrast identified small bowel involvement in the lateral side wall of the hematoma within the pelvis.

Computed tomography (CT) of the abdomen and pelvis showed moderate-to-large volume hemoperitoneum. The epicenter of the hemorrhage appeared to be in the pelvis where a rectovesical hematoma mass measured 8.2 × 7.0 cm (Fig. 1). The center of the hematoma raised suspicion for active hemorrhage consistent with contrast blush. No source of bleeding was identified on arteriography. Magnetic resonance imaging (MRI) with contrast ensued and suggested small bowel segment involvement in the lateral side wall of the hematoma within the pelvis (Fig. 2). Considering the patient presentation, imaging, and declining hemoglobin (Hb 8.3 g/dl), the patient was brought to the operating room for an exploratory laparotomy. Old blood was immediately encountered upon entering the abdomen during surgery. An exophytic mass, about 5 cm in diameter located on the antimesenteric border of the mid jejunum, was noted. The mass was actively oozing and was determined to be responsible for the hemoperitoneum. Specimens from the pelvic side wall, small bowel, and the hematoma were collected and sent to pathology. Small bowel resection with anastomosis was performed on the main tumor, though it was thought that residual disease was left. All segment pathology diagnoses showed over 5 cm with 11 mitoses per 5 mm^2^ GIST with associated blood clot, confirming stage 4 disease.

**Figure 3:**
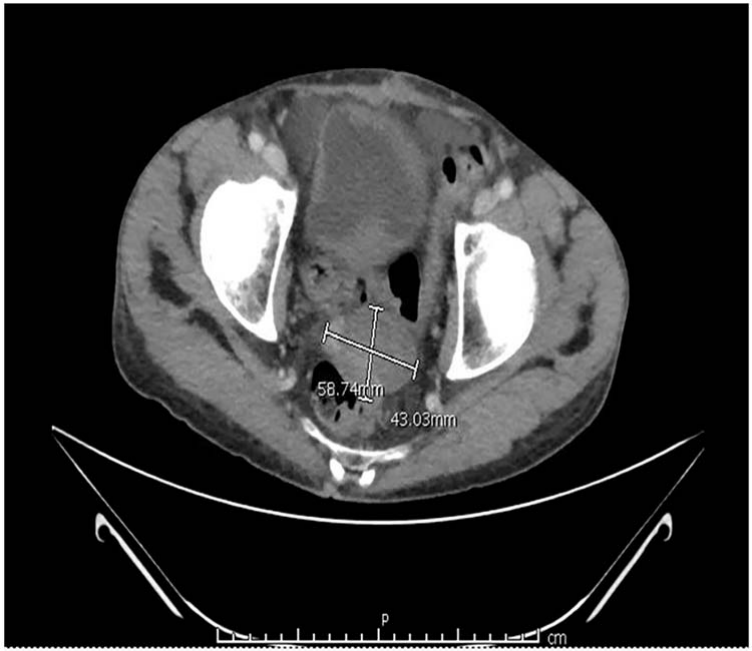
CT abdomen and pelvis with contrast identified small bowel distention and a mass-like focus measuring 5 × 3.8 cm in the pelvic region.

**Figure 4:**
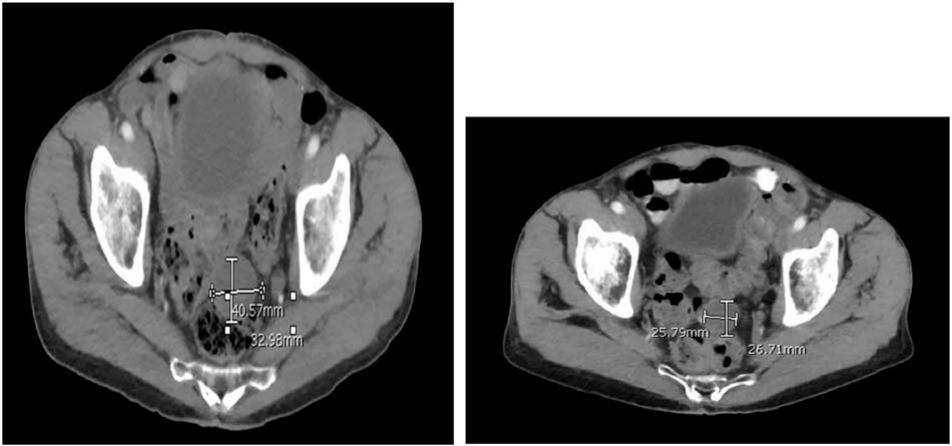
CT abdomen and pelvis at (**A**) 2- and (**B**) 4 months follow-up. The pelvic mass measured 4.1 × 3.3 and 2.6 × 2.7 cm, respectively.

Two months after the tumor was grossly resected and 1 month into Imatinib treatment, the patient presented to the Emergency Department with complaint of abdominal discomfort, constipation and nausea. CT abdomen and pelvis noted mid small bowel distention, suggestive of partial small bowel obstruction with mild ascites. Importantly, a mass-like focus measuring 5 × 3.8 cm was found in the pelvis anterior to the rectosigmoid junction (Fig. 3). A nasogastric tube was subsequently placed and the patient was administered empiric antibiotics. Considered a failure of conservative management, the patient was taken to the operating room again for an exploratory laparotomy, lysis of adhesions and debulking of the mass. The patient tolerated the procedure well with no complications. The patient maintained a typical postoperative course and was discharged with oncological follow-up and continued Imatinib treatment. On 2 and 4 months follow-up CT, the pelvic mass measured 4.1 × 3.3 and 2.6 × 2.7 cm (Fig. 4), respectively.

## DISCUSSION

The most frequent symptoms presented with GISTs are gastrointestinal bleeding, weight loss, and anemia. Patients may also present with acute abdomen secondary to obstruction, rupture, perforation, and peritonitis. In a study of 92 patients with GIST-related emergencies, Sorour *et al*. found gastrointestinal bleeding (48.91%) to be the most common symptom. Interestingly, the work reported that all 4 patients with incomplete resection (3 with extensive peritoneal deposits, 1 with a huge fixed mesenteric GIST) were not disease free by 3-year follow-up, which was the case with our patient. The 3-year disease free survival and overall survival of all patients were 73.2 and 92.1%, respectively [8]. Accordingly, complete GIST resection, though sometimes challenging, is vital for improved patient outcome.

Targeted therapy with tyrosine kinase receptor inhibitors such as Imatinib have been in use for the management of GISTs. Imatinib promotes apoptosis in GIST cells and inhibits cellular proliferation. Over 80% of patients with malignant or inoperable GISTs note a greater than 50% relief in tumor burden with Imatinib use. In addition, improved patient results can be noted as early as 4 weeks, where there was a 52% reduction in tumor size [9]. Our patient showed excellent response to Imatinib, evidenced by a continuously shrinking tumor on follow-up CT. In a study of eight patients with ongoing partial remission at the time of surgery treated with Imatinib, seven achieved histologically complete R0 resection while only one had disease progression [10]. Resultantly, patients with incomplete resection, such as ours, may achieve complete remission through successful therapy and follow-up.

The current literature reports 45 cases of ruptured small intestinal GISTs, with 31 (68.9%) presenting in males (Table 1). Like most cancers, GISTs presented more common in the elderly, though the range (22–87 years) was broad. GISTs tended to be quite large with diameters larger than 5 cm, and most commonly found at the jejunum.

**Table 1:** Reports of perforated gastrointestinal stromal tumors (GISTs) of the small intestine

**First author**	**Year**	**Sex/age**	**Location**	**Size (cm)**	**Intraoperative finding**	**Treatment**	**Follow-up (mo.) Survival**
Yamamoto *et al*. [11]	2003	M/32	NR	15	Peritonitis	SBR + Imatinib	24 alive
Ajduk *et al*. [12]	2004	F/60	Jejunum	7.5	NR	SBR	NR
Cegarra-Navarro *et al*. [13]	2005	M/76	Jejunum	6	NR	SBR	31 alive
Efremidou *et al*. [14]	2006	M/66	Ileum	7 × 5 × 4	Peritonitis	SBR + Imatinib	44 alive
Karagülle *et al*. [15]	2008	M/70	Jejunum	5	Abscess	SBR	13 alive
Hirasaki *et al*. [16]	2008	F/87	Ileum	13 × 11	NR	SBR	16 alive
Versaci *et al*. [17]	2009	M/46	Jejunum	12 × 7	Peritonitis	SBR + Imatinib	12 alive
Taniguchi *et al*. [18]	2009	M/59	NR	7.5	Peritonitis	SBR + Imatinib	14 alive
Licursi *et al*. [19]	2009	M/47	Jejunum	12.5 × 5	Peritonitis	SBR	NR
Ku *et al*. [20]	2010	F/33	Jejunum	6.5 × 5 × 4	Peritonitis	SBR	NR
Özben *et al*. [21]	2010	M/65	Ileum	8 × 5	Peritonitis	SBR + Ileostomy	NR
Varras *et al*. [22]	2010	F/28	NR	13	NR	SBR	36 alive
Feng *et al*. [23]	2011	M/45	Jejunum	10 × 8	Peritonitis	SBR	NR
Paramythiotis *et al*. [24]	2011	M/56	Jejunum	3	Peritonitis	SBR + Imatinib	48 alive
Bhandarwar *et al*. [25]	2011	F/55	Jejunum	9.5 × 8.5 × 7.5	Peritonitis	SBR	NR
Chen *et al*. [26]	2011	M/22	Jejunum	5	NR	SBR	2 alive
Aslan *et al*. [27]	2012	F/50	Jejunum	13	Peritonitis	SBR	NR
Memmi *et al*. [28]	2012	M/59	Jejunum	12	Peritonitis	SBR	NR
Choudhary *et al*. [29]	2012	M/35	Jejunum	4.5 × 3.5 × 2.5	Peritonitis	SBR	48 alive
Sezer *et al*. [30]	2012	F/61	Jejunum	5 × 2	Peritonitis	SBR + Imatinib	6 alive
Roy *et al*. [31]	2012	M/46	Jejunum	3 × 2	Peritonitis	SBR + Imatinib	6 alive
Beltrán *et al*. [32]	2013	M/46	Ileum	7.5 × 7	Abscess	SBR + Imatinib	NR
Nannini *et al*. [33]	2013	F/45	Jejunum	12	NR	SBR + Imatinib	13 alive
Shoji *et al*. [34]	2014	M/61	Jejunum	9 × 7	Peritonitis	SBR + Imatinib	36 alive
Misawa *et al*. [35]	2014	M/70	Jejunum	9 × 9	Abscess	SBR + Imatinib	12 alive
Sharma *et al*. [35]	2014	F/50	Ileum	10 × 8	Peritonitis	SBR + Imatinib	NR
Mansoor *et al*. [36]	2014	M/41	Multiple	NR	Peritonitis	SBR + Imatinib	NR
Alessiani *et al*. [37]	2015	M/82	Jejunum	7 × 5 × 4	Peritonitis	SBR + Imatinib	6 alive
Attaallah *et al*. [38]	2015	M/46	Jejunum	6 × 5.5	Peritonitis	SBR + Imatinib	NR
Cabral *et al*. [39]	2015	F/49	Jejunum	14	NR	SBR	NR
Jain *et al*. [40]	2016	M/65	Jejunum	6 × 5 × 3	Peritonitis	SBR + Imatinib	6 alive
Sagar *et al*. [41]	2016	M/60	Jejunum	7 × 5.5	NR	SBR + Imatinib	6 alive
Fukuda *et al*. [42]	2017	M/72	NR	2	NR	SBR + Imatinib	5 alive
Khuri *et al*. [43]	2017	F/69	Jejunum	9.5 × 6.4	NR	SBR	24 alive
Sato *et al*. [44]	2017	M/74	Jejunum	10 × 7	Peritonitis	SBR + Imatinib	22 dead
Prakash *et al*. [45]	2017	F/60	Ileum	6 × 8	Peritonitis	SBR + Imatinib	NR
Tajima *et al*. [46]	2018	M/75	Ileum	5.5	NR	SBR + Imatinib	9 alive
Takahashi *et al*. [47]	2019	M/64	NR	11	NR	SBR + Imatinib	18 alive
Arata *et al*. [48]	2020	M/46	Jejunum	7 × 6.5	Peritonitis	SBR + Imatinib	NR
Serban *et al*. [49]	2020	NR/71	Ileum	3 × 3.5 × 5	Peritonitis	SBR	NR
Al-swaiti *et al*. [50]	2020	M/59	Jejunum	11 × 9	Peritonitis	SBR + Imatinib	2 alive
Kimura *et al*. [51]	2020	M/46	Ileum	12 × 10 × 6	NR	SBR + Imatinib	168 alive
Meneses *et al*. [52]	2020	M/46	Jejunum	13 × 6 × 7.5	Peritonitis	SBR + Imatinib	NR
Tada *et al*. [53]	2021	M//77	Ileum	8	NR	SBR + Imatinib	3 alive
Senti *et al*. [54]	2021	F/50	Ileum	5.5 × 3	Peritonitis	SBR	30 alive

Cm: centimeter; NR: not reported; SBR: small bowel resection; Mo: months

## CONCLUSION

Considering the high recurrence rate of GISTs, management by surgical resection and post-operative Imatinib is recommended. Especially in patients with incompletely resected tumors, Imatinib induction has the potential to minimize disease progression and decrease recurrence.

## CONFLICT OF INTEREST STATEMENT

The authors declare no conflict of interest.

## FUNDING

None.
